# Trends of cervical tumours amongst women from perspectives of demographic, socioeconomic and geographic indicators: retrospective ecological study in Czechia

**DOI:** 10.3389/fpubh.2024.1347800

**Published:** 2024-05-15

**Authors:** Ondrej Holy, Ondrej Machaczka, Tereza Schovankova, Daniela Navratilova, Jarmila Zimmermannova, Romana Klasterecka, Jiri Vevoda

**Affiliations:** ^1^Science and Research Centre, Faculty of Health Sciences, Palacký University Olomouc, Olomouc, Czechia; ^2^Department of Healthcare Management and Public Health, Faculty of Health Sciences, Palacký University Olomouc, Olomouc, Czechia; ^3^Department of Preclinical Subjects, Faculty of Health Sciences, Palacký University Olomouc, Olomouc, Czechia; ^4^Department of Humanities and Social Sciences, Faculty of Health Sciences, Palacký University Olomouc, Olomouc, Czechia

**Keywords:** human papillomavirus, cervical tumour, women, vaccination rate, indicators

## Abstract

**Introduction:**

For many infectious diseases, women are at higher risk and have a more severe disease course than men for many reasons, including biological differences, social inequalities, and restrictive cultural norms. The study focuses on infections with human papillomaviruses (HPV) in the form of cervical cancer as a gender-specific disease. The main goal is to evaluate cervical tumour incidence trends in the Czech female population in the HPV vaccination period 2012–2020 in relation to selected demographic, socioeconomic, and geographic indicators.

**Methods:**

This is a retrospective ecological study. Data from publicly available databases about the incidence and mortality of cervical tumours (C53 Malignant neoplasm of cervix uteri, D06 Carcinoma *in situ* of cervix uteri according to ICD 10) and HPV vaccination rate were analysed and compared with demographic, socioeconomic and territorial data. Associations were searched using correlation analysis.

**Results:**

There was a decreasing trend in the incidence of cervical cancer in the observed period. Regarding cervical tumours (C53, D06) and malignant neoplasm of cervix uteri incidence (C53), the decrease was approximately 11 and 20%, respectively. Differences between regions were observed in incidences and vaccination rates. Based on correlation analysis, indicators connected with urban/rural aspects, such as a share of urban population and population density, were statistically significant. The indicators related to higher cervical cancer incidence are the high unemployment rate of women, the high number of divorces, the high number of abortions, the high share of the urban population, the high number of students, and the high number of women with only primary education. On the other hand, the indicators related to lower cervical cancer incidence are the high gross domestic product (GDP), the high average gross monthly wage per employee, the high employment rate of women, the higher average age of mothers at birth, and the high number of women with tertiary education.

**Conclusion:**

Results underline the problem of economically disadvantaged regions and families. Increasing vaccination rates, promoting regular screening for cervical cancer, and supporting awareness in the population, especially in regions with higher incidence rates, should be priorities for public health efforts.

## 1 Introduction

Diseases are not only biophysical phenomena but have social and cultural causes and consequences. These also include the availability and nature of treatment and the extent to which treatment is accepted or adhered to. Currently, the emphasis is on an interdisciplinary approach to understanding health and disease. At the same time, more and more attention is being paid to the influence of gender on health status, as well as to the individual’s approach to disease prevention. For the purpose of this study, the terms “female” and “woman” are perceived from both a biological (sex) and sociological (gender) point of view. Gender refers to the cultural characteristics and models assigned to the male or female biological sex and refers to social differences between women and men (1).

Also, in infectious diseases, sexual dimorphism has been described (2). For many infectious diseases, women are at higher risk and have a more severe disease course than men (3). Health disbalance in infectious diseases between men and women is the result of interactions between biological and sociocultural factors, such as sex hormones, genetic predisposition, lifestyle, age, social inequalities, restrictive cultural norms, the geographic distribution of pathogens, and access to healthcare or comorbidities (3–5). Women are less burdened than men when it comes to developing most infectious diseases because of hormonal and chromosomal control of immunity (6). Estradiol provides immune protection, but progesterone and testosterone suppress anti-infective responses. Women demonstrate a more remarkable ability to recognise pathogens, recruit more innate immune cells, and mount stronger adaptive immune responses than men (5). Although the finding is that estradiol helps women manage infectious diseases, it is also necessary to be aware of the mentioned socioeconomic influences on the course of diseases (5). Women make up the dominant part of the population at risk of poverty, especially single mothers and pensioners. However, the social benefits system does not sufficiently consider this aspect. The consequence of infectious disease is that it will restrict various areas of a woman’s life. They can only occur for a certain period, long-term or permanently, while some symptoms accompanying the disease can also negatively affect the work sphere. There can be various complications that lead to long-term incapacity for work, which is related to the financial impact of illnesses. Socioeconomic factors, both material and psychosocial, can impact infectious diseases.

Regarding infectious diseases and their impact on the female population, the following work focuses on infections with human papillomaviruses (HPV) in the form of cervical cancer as a gender-specific disease. Worldwide, cervical cancer is the fourth most common cancer in women (7). HPV is the most common sexually transmitted infection. Before age 50, genital HPV infection occurs in 80% of women and at least 50% of men (8). HPV causes asymptomatic infections in most cases but also several benign diseases with high morbidity and several other premalignant diseases and cancers in both women and men. Cervical cancer is by far the most common HPV-related disease. About 99.7% of cervical cancer cases are caused by persistent genital high-risk HPV infection (9, 10). Regarding scientific studies focused on cervical cancer and connected issues, there is still a gap. There exist studies dealing with trends of cervical cancer incidence and possible indicators which can influence both incidence and mortality.

One of such indicators is screening, vaccination, and the age of screening and vaccination. Screening and treatment of pre-cancer lesions with HPV vaccination are effective measures to eradicate cervical cancer as a global public health problem (11). According to Cancer Research United Kingdom (UK) (12), girls who are vaccinated between the ages of 12 and 13 have an 87% lower incidence of cervical cancer in their 20s compared to those who have not been vaccinated. The effectiveness decreased with the advanced age of the vaccinated. Also, vaccination of boys and men may reduce the incidence of cervical cancer and its precursors via herd immunity (13). Although there is no evidence of a clear impact on cervical cancer elimination by vaccinating boys, vaccination directly protects men from HPV-related diseases, and most high-income countries have implemented gender-neutral programmes. European Cancer Organization aims to have a gender-neutral approach all over Europe by 2030 (14). On the other hand, it is assumed that HPV vaccination rates declined as a result of the COVID-19 pandemic. According to the WHO, vaccination coverage worldwide decreased by over a quarter compared to 2019 (15).

Other important indicators are demographic and/or socio-economic factors. For example, a study from India (16) underlines the following significant risk factors for HPV infection: early age at marriage, lack of education, increased parity, early age at first pregnancy, poor sanitation, use of tobacco, and belonging to below poverty line. Buskwofie et al. (7) observed the situation in the USA and depicted the following risk factors: racial and ethnic minorities and socioeconomically disenfranchised. Concerning the situation in China, the influence of HPV-related knowledge on HPV testing also lies in the joint effects of socio-demographic factors, including residence, education, and monthly income (17). Besides the factors mentioned above, there are others, such as location and/or differences between urban and rural areas (7, 18, 19).

Concerning the situation in Czechia, roughly 92% of cervical, 35% of vulvar, 82% of anal and 65% of oropharyngeal tumours were associated with HPV types included in the nonvalent HPV vaccine (20). Currently, three prophylactic vaccines against HPV infection are available: bivalent Cervarix, quadrivalent Gardasil (formerly Silgard) and nonavalent Gardasil9. The insurance companies have covered HPV vaccination for girls aged 13 since 2012 and boys of the same age since 2018. According to available vaccination data, the vaccination rate of girls aged 13 represented 75.7% in 2012 and only 60.2% in 2018 (21). Currently, the vaccination rate is around 60; on the other hand, there are significant differences between regions (21). The main cause of insufficient vaccination in Czechia is “vaccine hesitancy,” the distrust in vaccination caused by the spread of misinformation (22). On the other hand, the significant increase in the number of vaccinated boys, which was only 29.7% in 2018/19, is positive (23).

EUROSTAT states that cervical cancer screening coverage is 52.5% (24). All Czech women over 15 years old are screened yearly by Pap test. From 2021, the HPV screening test (examination of the presence of nucleic acid of high-risk types of HPV in cervical smear) is paid by public health insurance funds for all women aged 35 and 45. The overall prevalence of HPV in Czechia remains relatively high, with a 2020 study (24, 25) about 6.6% of women in the general population are estimated to harbour cervical HPV-16/18 infection and 79.3% of invasive cervical cancers are attributed to HPVs 16 or 18. According to the HPV information centre and its estimation for 2020 (24), about 769 new cervical cancer cases are diagnosed, and about 398 cervical cancer deaths occur annually in Czechia.

The main goal of this paper is to evaluate the incidence trends of cervical tumours in the Czech female population in the HPV vaccination period 2012–2020 in relation to selected demographic, socioeconomic, and geographic indicators. The sub-goals were to analyse: (i) the trends in the incidence and mortality of cervical tumours over the vaccination period 2012–2020; (ii) the differences in the cervical tumours incidence rate between regions, urban and rural areas; (iii) relationship of the cervical tumours incidence rate with selected demographic and socioeconomic indicators.

Unique is that this paper focuses on not only malignant cervical tumours but also carcinoma *in situ*. The incidence of all these cervical tumours (both malignant neoplasm C53 and carcinoma *in situ* D06 according to ICD 10) more closely reflects the risk of HPV exposure. Also, the unique location of Czechia in the centre of Europe and its regional diversity enables the transferability of the results of our study to other regions as well. Therefore, this data analysis that our study will bring could be used for nationwide education regarding HPV knowledge. The new perspectives on the issue of HPV, which our study offers, can significantly contribute to the development of knowledge in this area and thus support the prophylaxis of this type of disease not only in the female population but all over the world.

## 2 Materials and methods

### 2.1 Study settings

This is a retrospective ecological study based on analysis of cervical tumours (C53, D06) incidence trends in relation to demographic and socioeconomic indicators. The study population was women from Czechia between 2012 and 2020. The datasets used and/or analysed during the current study are all publicly available.

### 2.2 Study location

Czechia (the Czech Republic) is a country in Central Europe with a population of 10,516,707, of which 5,332,932 are women (26). The average life expectancy for women was 82.1 years in 2019 (27). Women population at risk for cervical cancer C53 (female population aged > = 15 years) is about 4.6 million (24). According to estimations for 2020, cervical cancer ranks as the 11th leading cause of female cancer and as the 8th leading cause of cancer deaths of female cancer deaths in Czechia (24). Czechia is divided into 14 regions, which are: Prague, the Capital City (PCC), Central Bohemia Region (CBR), South Bohemian Region (SBR), Plzeň Region (PLR), Karlovy Vary Region (KVR), Ústí nad Labem Region (ULR), Liberec Region (LBR), Hradec Králové Region (HKR), Pardubice Region (PAR), Vysočina Region (VYR), South Moravian Region (SMR), Olomouc Region (OLR), Zlín Region (ZLR), Moravian-Silesian Region (MSR). Furthermore, only abbreviations of regions are used for the purpose of this study.

### 2.3 Input data

All the data comes from the State Statistical Service, which acquires data and compiles statistical information on Czechia’s social, economic, demographic, and ecological development. The primary data sources were the State Statistical Service authorities such as the Czech Statistical Office (CSO) and The Institute of Health Information and Statistics of the Czech Republic (IHIS), which administrates the National Health Information System. These authorities are governed by principles of the European Statistics Code of Practice. Data about women in 5-year age categories (age 0–85+) at the level of 14 regions of the Czechia for the period 2012–2020 was used. The following population data from publicly available databases was used as input data sources.

#### 2.3.1 Health data

##### 2.3.1.1 Incidence and mortality of cervical tumours

Absolute incidence and mortality of cervical tumours (C53 Malignant neoplasm of cervix uteri, D06 Carcinoma *in situ* of cervix uteri according to ICD 10) were obtained from the National Oncological Register administered by the IHIS and processed by the Institute of Biostatistics and Analyses (28).

##### 2.3.1.2 HPV vaccination rate

HPV vaccination rate was obtained from the National Register of Reimbursed Health Services administered by the IHIS. Vaccination against HPV is identified from the documents on reported health care using the ATC code J07BM or one of the procedures 02110, 02125 in combination with diagnosis Z258. HPV vaccination rate of prime-vaccinated female patients relative to the female population aged 13 years between 2012 and 2019 is presented (the number of females vaccinated in a given year corresponds to patients who reached the age of 13 in a given year and were vaccinated in a given or the following calendar year). The insurance companies have covered HPV vaccination for girls aged 13 since 2012 in Czechia.

#### 2.3.2 Demographic and socioeconomic data

Demographic and socioeconomic data were obtained exclusively from the CSO. The data used are freely available and aggregated at the level of regions of Czechia. These data can be divided into:

- Data about the age distribution of the women population published yearly (29).
- Data about territorial comparison of demographic and socioeconomic indicators by regions, which concern the entire population (30). They contain indicators that are not gender specific. In correlation, there were used indicators related to the whole population about:
  - population (population density, share of urban population, total population change, infant mortality, number of students)
  - migration (immigration, emigration)
  - socioeconomic indicators (gross domestic product, average gross monthly wage per employee, pension recipients, number of old-age pensions)
- Data about territorial comparison of demographic and socioeconomic indicators by regions, which concern only women (31). There were used indicators about:
  - age
  - population gain/loss (total and natural population gain/loss, births, deaths)
  - marriages and divorces
  - abortions
  - level of education
  - employment (employment and unemployment rate).

#### 2.3.3 Geographical data

The layer Boundaries from the Topographic database of the Czech Republic (Data200) were used. The database is published under a Creative Commons CC BY 4.0 licence by the State Administration of Land Surveying and Cadastre (32).

### 2.4 Data processing

#### 2.4.1 Conversion of absolute incidence and mortality to relative numbers (per 100,000 women)

The relative incidence and mortality numbers were calculated from absolute values according to the average state of the population as of the first of July of the given year according to the data from CSO. These indicators were calculated by dividing the published number by the population size for each region, and each age group was displayed as units available for 100,000 women. Furthermore, only these relative numbers were used.

#### 2.4.2 Analysis of trends in the incidence and mortality of cervical cancer

Trends of incidence and mortality of cervical cancer were analysed during the monitored period, and a sub-analysis of age distribution in 5-year age categories (age 0–85+) was made. First, the analysis was conducted for Czechia overall and subsequently for individual regions. Relative incidence and mortality trends in the individual regions were compared with the overall trend of Czechia using correlation analysis.

#### 2.4.3 Spatial visualisation and identification of the regions with the lowest and highest incidence

The data was visualised through analytical maps and colour scales to determine the spatial phenomenon. The maps were created using QGIS 3.26.3 software. The regions with the highest and lowest incidence were selected for the following analysis based on the data.

#### 2.4.4 Analysis of the incidence of cervical cancer in relation to demographic, socioeconomic and geographic indicators in regions

Associations of incidence of cervical cancer with selected demographic and socioeconomic indicators (specified in Input Data) were searched using correlation analysis. The evolution of year incidence during the studied period (dependent variable *Y*) was compared with the trend of each regional socioeconomic and demographic characteristic specified in Input Data (independent variable *X*). Correlations were calculated for selected regions only. Pearson correlation coefficient was calculated using TIBCO Statistica software. The study did not include variables that were correlated or a subset of another variable. This was tested using the Correlation matrix. Statistical significance cut-off was determined at *p* < 0.05.

## 3 Results

### 3.1 Trends in the incidence and mortality of cervical tumours

**Figure 1:**
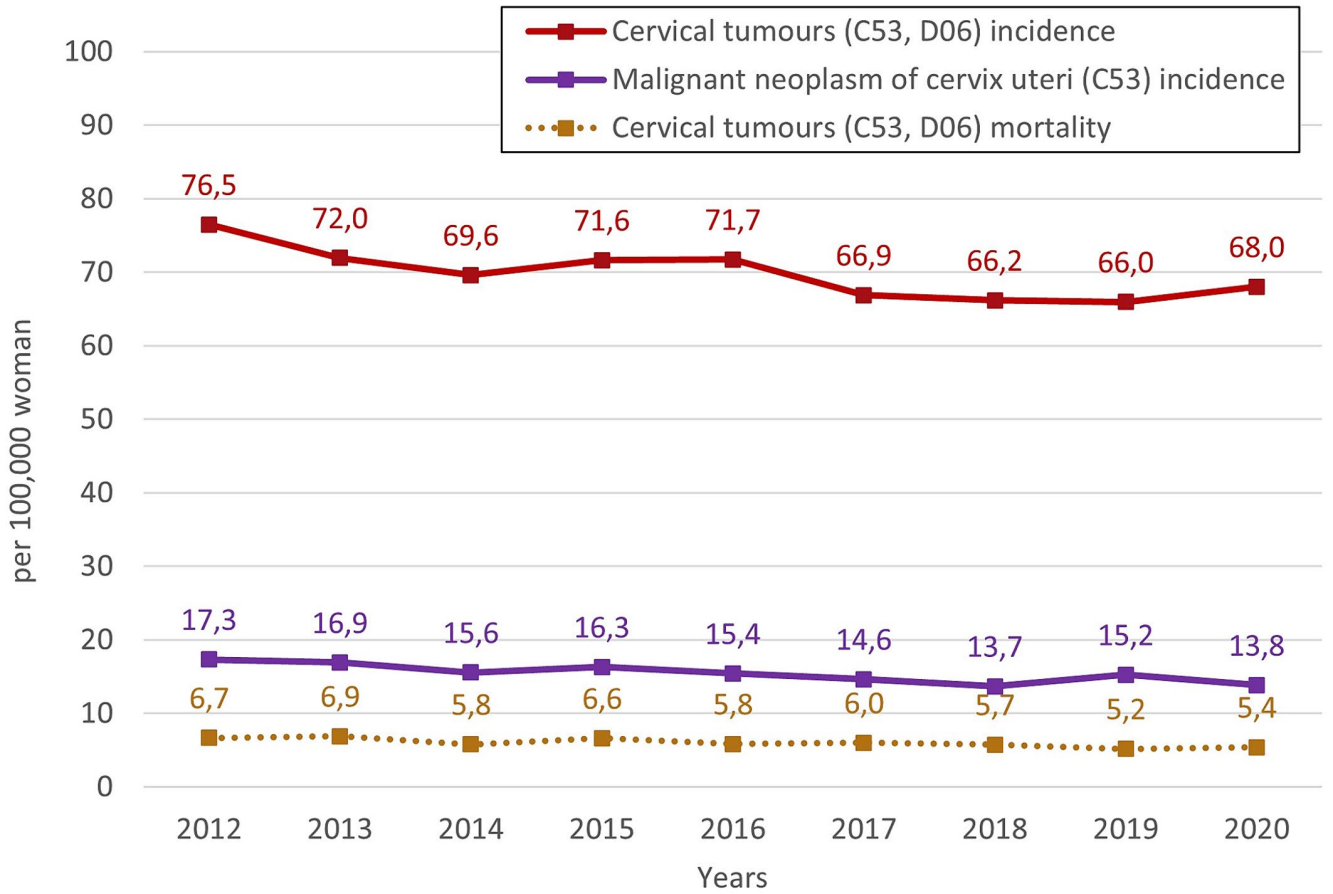
Overall trends of the relative incidence and mortality of cervical tumours in Czechia for the monitored period 2012–2020.

Figure 1 shows overall trends of the relative incidence and mortality of cervical tumours in Czechia for the monitored period from 2012 to 2020. There is a noticeable overall decreasing trend in cervical tumours (C53, D06) incidence, only with some higher incidences in years 2015 and 2016. In the last year of the studied period, the incidence increased slightly. The relative incidence of cervical tumours (C53, D06) in 2020 has decreased by 11.07% compared to 2012. In the case of separate malignant neoplasm of cervix uteri (C53) incidence, the overall trend is slightly decreasing, with some higher incidence in 2015 and 2019. However, the relative incidence in 2020 has decreased by 20.25% compared to 2012. Relative mortality of cervical tumours (C53, D06) is persistently low with a slightly decreasing trend, and in 2020, it decreased by 19.10% compared to 2012. This mortality is caused only by malignant neoplasm of cervix uteri (C53). So cervical tumours (C53, D06) mortality, which is used further, is equal to the separate mortality of C53.

**Figure 2:**
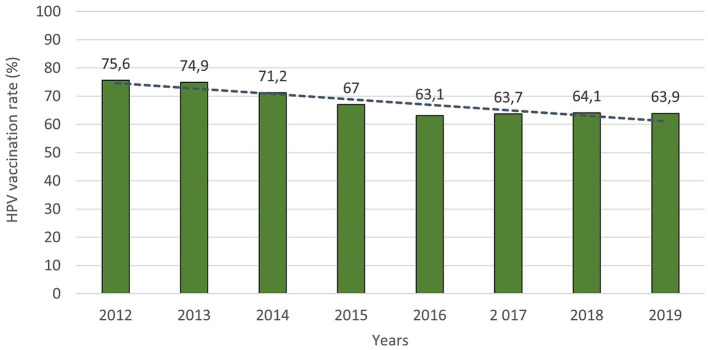
HPV vaccination rate of prime-vaccinated female patients relative to the female population aged 13 years between 2012 and 2019 (the number of females vaccinated in a given year corresponds to patients who reached the age of 13 in a given year and were vaccinated in a given or the following calendar year).

**Figure 3:**
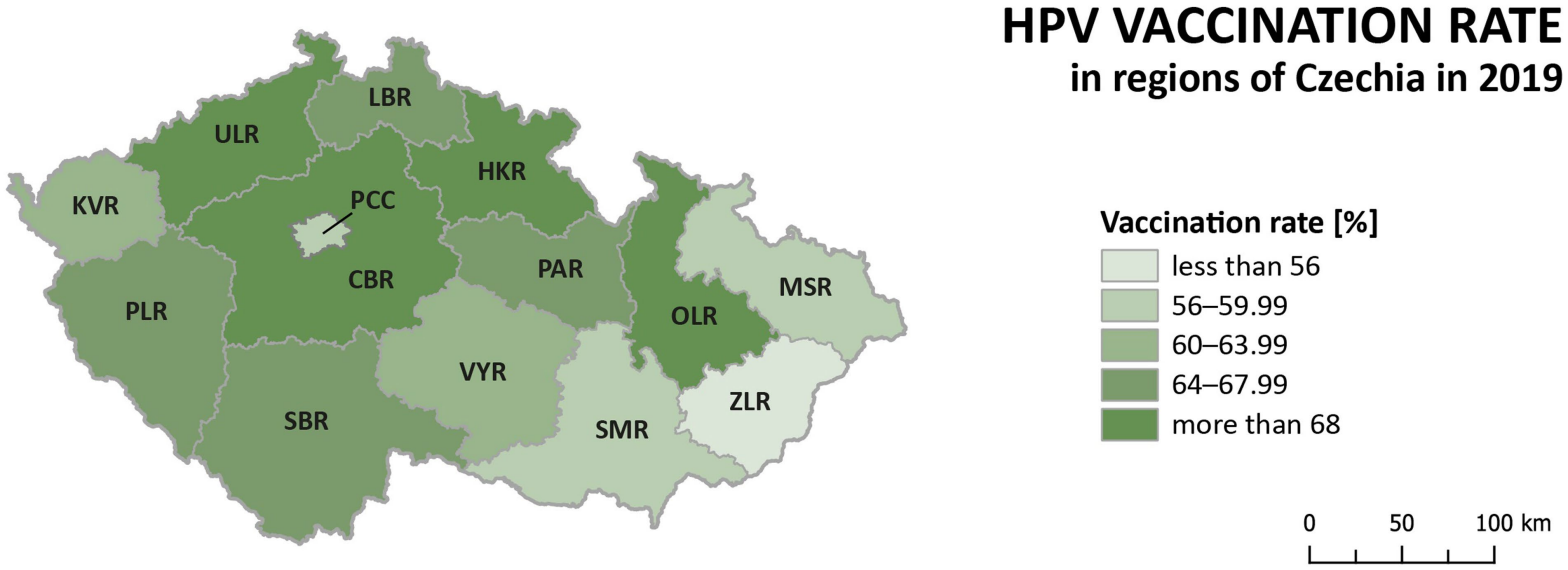
HPV vaccination rate of prime-vaccinated female patients relative to the female population aged 13 years in regions of Czechia in 2019 (the number of females vaccinated in a given year corresponds to patients who reached the age of 13 in a given year and were vaccinated in a given or the following calendar year).

The HPV vaccination rate of prime-vaccinated female patients relative to the female population aged 13 between 2012 and 2019 in Czechia is shown in Figure 2. The number of females vaccinated in a given year corresponds to patients who reached the age of 13 in a given year and were vaccinated in a given or the following calendar year. Data about the HPV vaccination rate for the year 2020 was not published at the time of processing this paper. There has been a noticeable decrease in the vaccination rate from the start of vaccination in 2012 to 2019. Vaccination rates fell by 11.7% between those years. Figure 3 shows the HPV vaccination rate of particular regions in 2019. Differences between individual regions are evident from this visualisation. There are regions where the vaccination rate is less than 60% (ZLR, MSR, SMR, PCC).

Table 1 shows the relative incidence of cervical tumours in the studied period and age distribution analysis. All cervical tumours (C53, D06) were most often diagnosed in the age group 20–44 years. The incidence was higher than 100 per 100,000 women in these age groups. The highest incidence was in the age group 25–34 years. In separate malignant neoplasm of cervix uteri (C53), the higher incidence (over 20 per 100,000 women) started appearing from the age group 35–39 years and above. Table 2 shows the relative mortality of cervical tumours (C53, D06) in the studied period and age distribution analysis. Mortality was most frequent in the older age groups. It increased significantly from over 60 years with the highest frequency in age over 85.

**Table 1:** Relative incidence of cervical tumours by age group in Czechia (2012–2020).

	Age	Incidence per 100,000 women of each age group								
		2012	2013	2014	2015	2016	2017	2018	2019	2020
Malignant neoplasm (C53) and carcinoma *in situ* (D06) of cervix uteri	0–4	0	0	0	0	0	0	0	0	0
	5–9	0	0	0	0	0	0	0	0	0
	10–14	0	0	0	0	0	0	0.4	0	0
	15–19	11.7	11.2	13.1	9.4	13.9	11.6	4.0	3.5	2.6
	20–24	**105.7**	**104.3**	**100.3**	98.5	98.8	94.2	97.0	72.1	59.1
	25–29	**215.6**	**198.3**	**195.1**	**199.4**	**202.8**	**187.4**	**190.5**	**150.9**	**161.4**
	30–34	**192.0**	**173.0**	**183.0**	**190.2**	**199.3**	**185.9**	**187.5**	**188.6**	**207.0**
	35–39	**152.2**	**157.6**	**139.6**	**152.1**	**142.4**	**139.5**	**139.4**	**147.2**	**167.5**
	40–44	**124.2**	**103.3**	**105.0**	**110.3**	**116.2**	**106.2**	**109.2**	**113.7**	**117.5**
	45–49	77.6	79.0	70.9	75.0	82.6	72.6	83.1	99.4	99.8
	50–54	45.2	44.6	40.4	51.8	47.6	45.5	43.6	55.0	60.4
	55–59	41.2	38.5	38.3	31.8	37.1	32.9	37.3	38.5	35.5
	60–64	35.4	38.7	37.1	35.5	37.1	37.5	34.5	31.2	29.0
	65–69	38.2	37.1	43.4	38.9	32.7	32.2	30.9	32.2	34.3
	70–74	31.9	31.4	27.2	32.6	35.1	30.3	26.4	38.0	31.3
	75–79	35.7	30.1	26.0	29.5	27.3	31.8	20.5	30.8	25.4
	80–84	32.6	26.1	26.3	36.6	28.5	23.4	17.3	17.0	25.8
	85+	36.3	26.9	28.3	22.8	19.2	29.4	22.6	21.0	20.3
	Total	76.5	72.0	69.6	71.6	71.7	66.9	66.2	66.0	68.0
Malignant neoplasm of cervix uteri (C53)	0–4	0.0	0.0	0.0	0.0	0.0	0.0	0.0	0.0	0.0
	5–9	0.0	0.0	0.0	0.0	0.0	0.0	0.0	0.0	0.0
	10–14	0.0	0.0	0.0	0.0	0.0	0.0	0.4	0.0	0.0
	15–19	0.8	0.0	0.0	0.4	0.4	0.0	0.4	0.0	0.0
	20–24	2.2	2.8	2.3	1.7	1.8	1.5	1.2	3.4	0.9
	25–29	8.6	7.9	8.8	12.1	6.8	5.4	8.8	8.1	5.2
	30–34	16.1	16.6	14.9	13.9	13.8	13.3	13.7	15.4	13.2
	35–39	**22.6**	**25.5**	**22.6**	18.3	18.1	17.6	15.7	17.6	**20.8**
	40–44	**29.5**	**23.3**	**20.2**	**23.5**	**22.5**	19.8	18.7	19.5	17.9
	45–49	**24.5**	**25.3**	**22.9**	**20.7**	**24.7**	**22.3**	**22.1**	**26.1**	**22.7**
	50–54	**20.4**	19.9	16.4	**23.1**	**25.0**	18.1	19.3	**24.0**	**21.3**
	55–59	**23.4**	**25.7**	**23.0**	**21.1**	**19.5**	**23.3**	**21.2**	**22.9**	18.8
	60–64	**23.0**	**27.7**	**23.4**	**25.2**	**24.2**	**24.9**	**21.9**	19.2	17.7
	65–69	**28.7**	**25.4**	**27.3**	**29.3**	**23.1**	**22.2**	19.4	**20.9**	**21.0**
	70–74	**23.4**	**22.1**	**20.8**	**21.7**	**23.8**	**21.9**	19.0	**27.8**	**20.2**
	75–79	**29.8**	**23.5**	**20.1**	**23.7**	**20.2**	**20.7**	16.4	**21.8**	17.2
	80–84	**28.7**	**20.2**	**24.3**	**29.3**	**23.8**	17.9	15.2	10.9	20.5
	85+	**32.9**	**26.9**	**24.4**	**22.1**	17.0	**25.1**	19.1	16.8	18.2
	Total	17.3	16.9	15.6	16.3	15.4	14.6	13.7	15.2	13.8

Bold text visualise age groups with highest incidence (C53, D06 incidence over 100 per 100,000 women, C53 incidence over 20 per 100,000 women).

**Table 2:** Relative mortality of cervical tumours by age group in the Czechia (2012–2020).

Age	Mortality per 100,000 women of each age group								
	Malignant neoplasm (C53) and carcinoma *in situ* (D06) of cervix uteri								
	2012	2013	2014	2015	2016	2017	2018	2019	2020
0–4	0	0	0	0	0	0	0	0	0
5–9	0	0	0	0	0	0	0	0	0
10–14	0	0	0	0	0	0	0	0	0
15–19	0	0	0	0	0	0	0	0.4	0
20–24	0	0.3	0.6	0	0	0.8	0	0	0
25–29	0.3	1.7	0.6	0.9	1.8	0	0.3	0.6	0.7
30–34	1.0	2.3	2.2	1.1	2.0	1.4	1.1	1.1	1.2
35–39	4.2	2.2	2.4	2.7	1.4	2.7	2.1	1.1	1.9
40–44	4.5	3.5	4.1	4.6	6.0	4.2	1.8	3.5	3.2
45–49	9.5	8.4	7.7	5.6	5.2	5.9	5.7	4.1	6.2
50–54	6.0	8.9	6.5	8.4	7.6	7.6	5.6	8.6	5.3
55–59	9.0	8.7	**11.1**	9.2	7.7	8.6	8.4	**10.0**	**10.0**
60–64	11.9	**12.3**	7.9	**14.3**	**10.7**	**10.1**	**11.8**	9.9	9.5
65–69	**14.0**	**14.8**	**12.5**	**12.0**	**12.3**	**12.7**	**13.4**	**10.7**	**11.6**
70–74	**21.3**	**15.7**	**11.7**	**21.4**	**11.4**	**15.8**	**15.0**	**12.3**	**14.0**
75–79	**12.5**	**21.9**	**17.4**	**14.2**	**16.7**	**11.1**	**13.2**	**12.8**	9.8
80–84	**23.4**	**17.6**	**13.8**	**20.0**	**16.3**	**16.5**	**17.3**	**10.2**	**12.6**
85+	**24.5**	**29.3**	**20.4**	**18.3**	**17.7**	**23.0**	**20.5**	**11.9**	**17.5**
Total	6.7	6.9	5.8	6.6	5.8	6.0	5.7	5.2	5.4

Bold text visualise age groups with highest mortality (C53, D06 incidence over 100 per 100,000 women, C53 incidence over 20 per 100,000 women).

### 3.2 Differences in the cervical tumours incidence and mortality between regions

**Figure 4:**
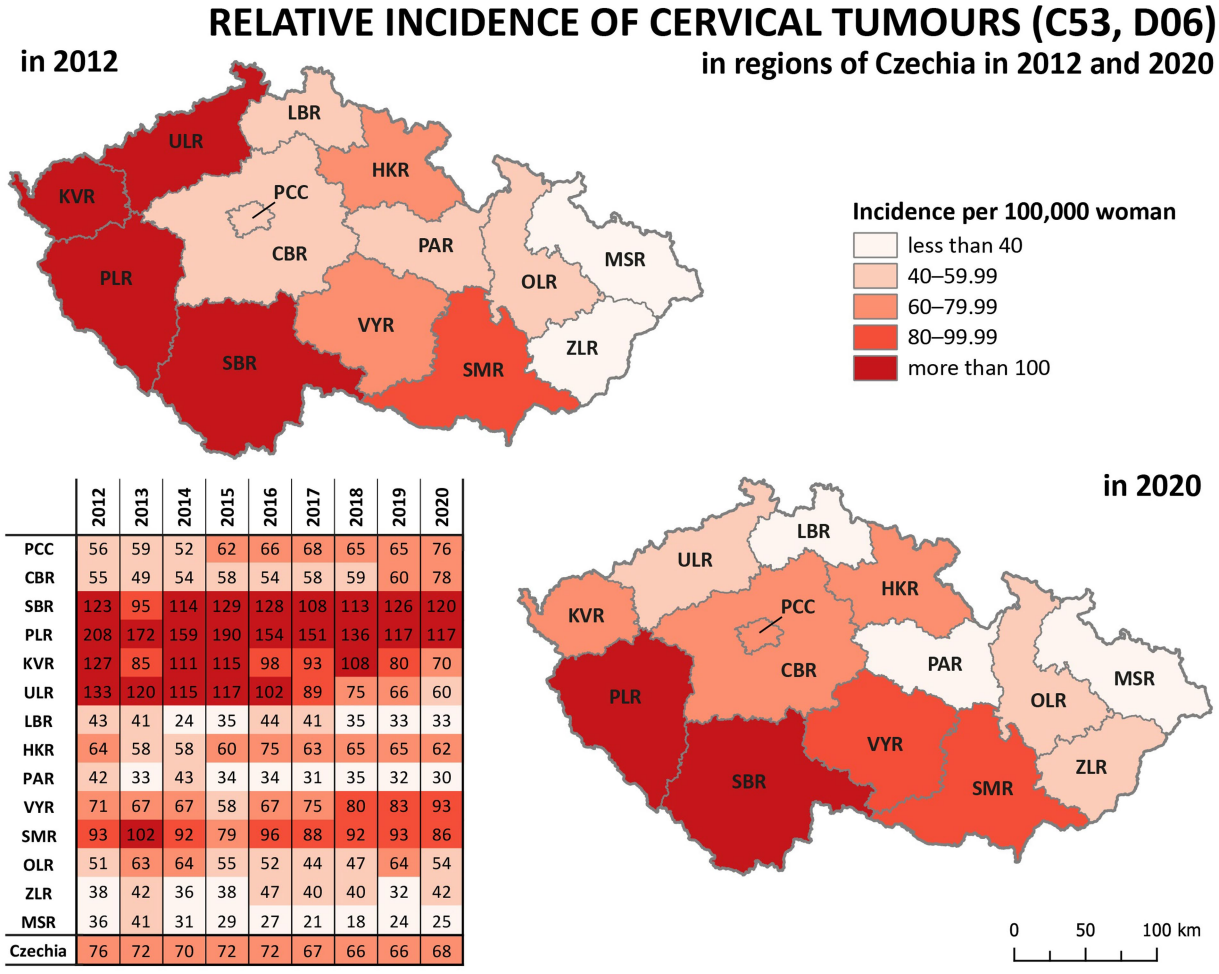
Comparison of cervical tumours (C53, D06) relative incidence in 14 regions of the Czechia (2012–2020).

**Figure 5:**
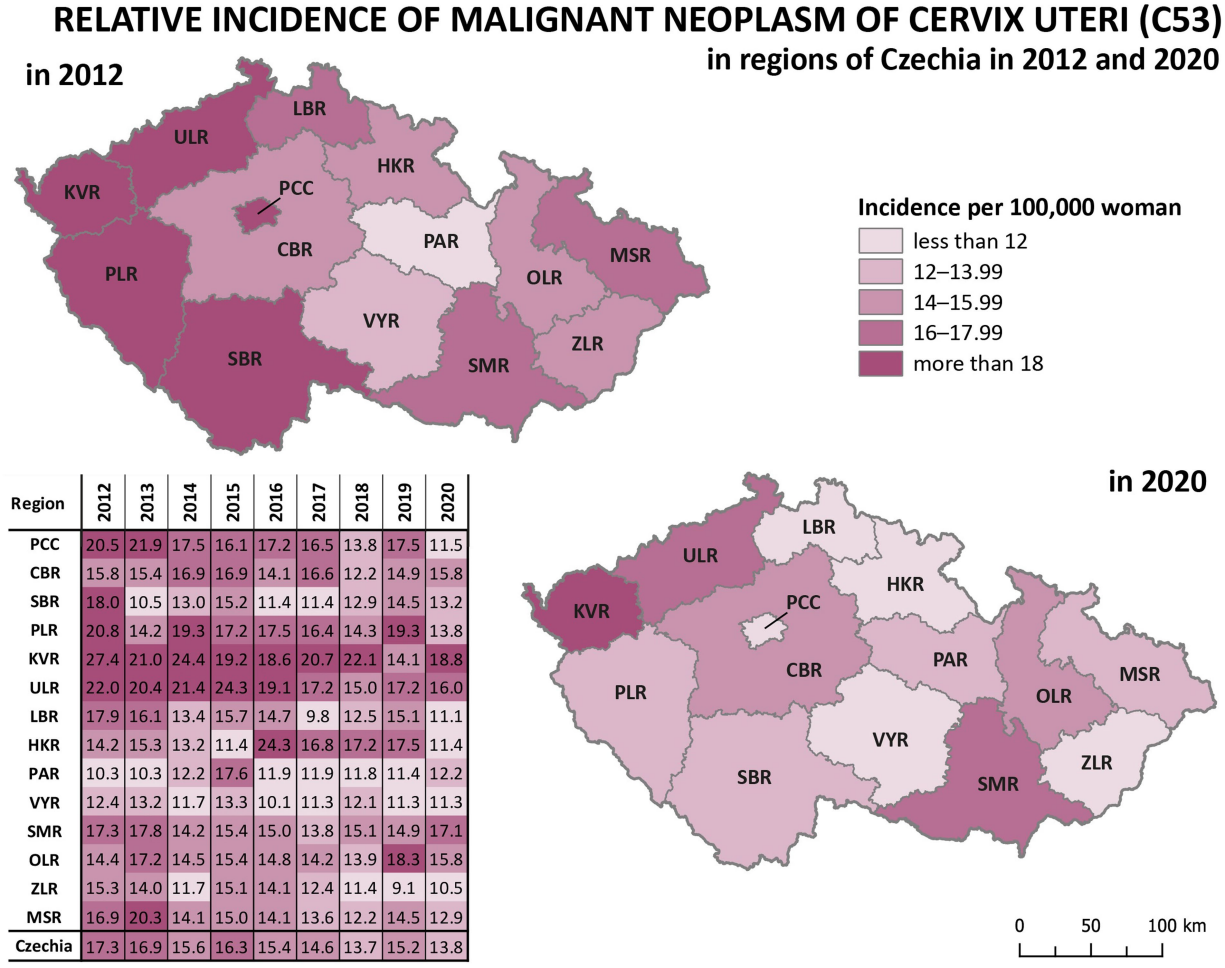
Comparison of malignant neoplasm of cervix uteri (C53) relative incidence in 14 regions of Czechia (2012–2020).

**Figure 6:**
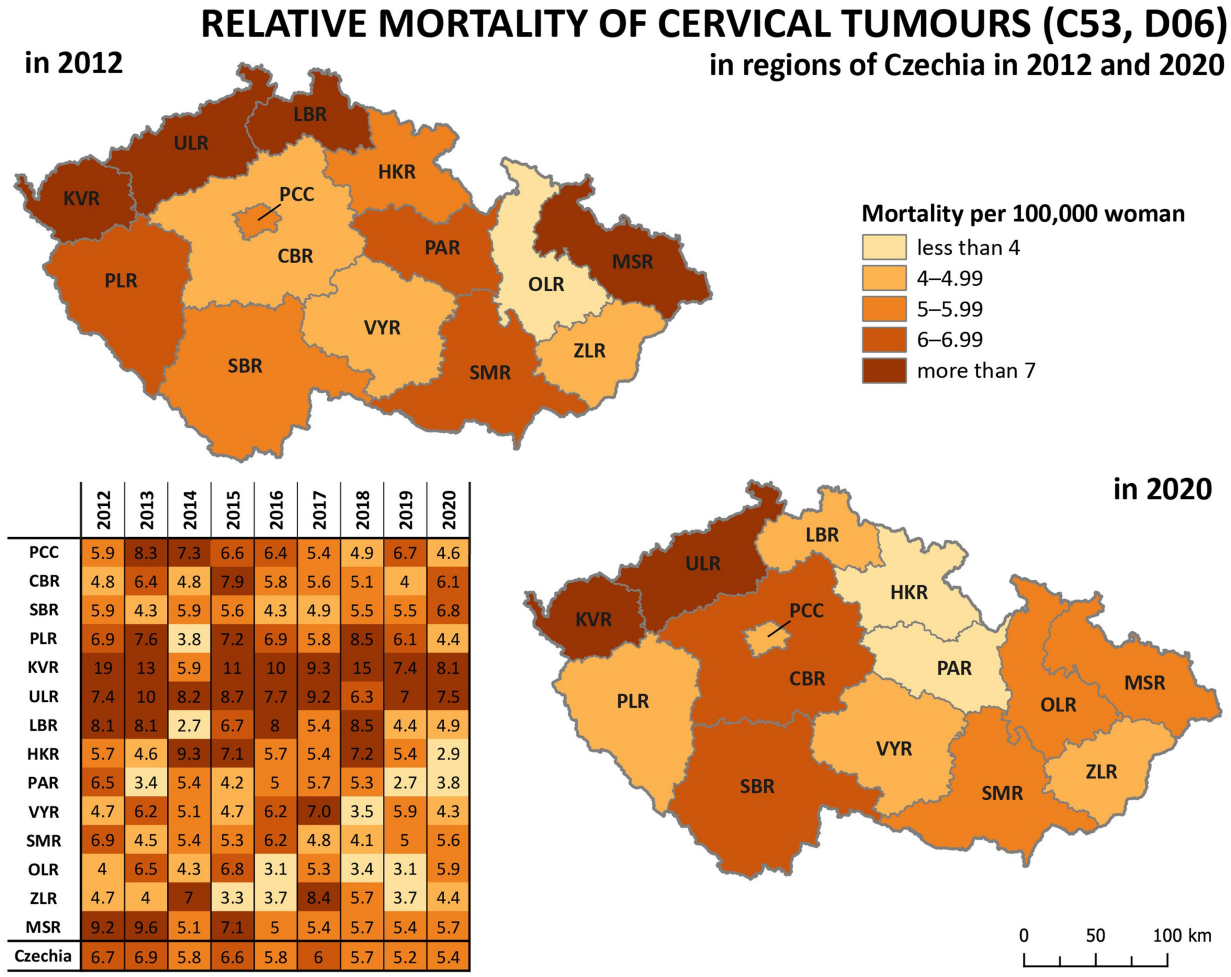
Comparison of cervical tumours (C53, D06) relative mortality in 14 regions of Czechia (2012–2020).

Analysis of relative incidence trends in 14 regions of Czechia for cervical tumours (C53, D06) is shown in Figure 4 and separately for malignant neoplasm of cervix uteri (C53) in Figure 5. The data are visualised in the form of analytical maps for the initial and final years (2012 and 2020) and by colour scales for all studied years. Table 3 shows the correlation between cervical tumours’ relative incidence and mortality for each region and the overall trend of Czechia. In the case of all cervical tumours (C53, D06), the difference in the individual regions is evident, even in trends through the studied period (Figure 4). As in the whole of Czechia, there is a decrease in incidence in some regions, but there are also regions where, on the contrary, there is a slight increase (PCC, VYR, CBR). Regions PLR and MSR constantly had the highest and lowest incidence, respectively, in all studied years. For both regions, the trend is correlated with the decreasing overall trend of Czechia, as shown in Table 3. There are no evident regions with consistently opposite incidence numbers in separate malignant neoplasms of the cervix uteri (C53) (Figure 5). There are differences in the individual regions. However, there are no apparent persistent trends throughout the studied period. A comparison of cervical tumours (C53, D06) mortality trends is shown in Figure 6. Higher relative mortality (over 7 per 100,000 women) is apparent in KVR and ULR regions and is persistently higher in almost all studied years. From the other point of view, the comparison does not show a region with significantly lower mortality, which would be stable through the analysed period.

**Table 3:** Correlation of relative incidence and mortality in regions with the overall trend of Czechia (2012–2020).

Regions of Czechia	Cervical tumours (C53, D06) incidence of Czechia	Malignant neoplasm cervix uteri (C53) incidence of Czechia	Cervical tumours (C53, D06) mortality of Czechia
PCC	−0.52112	0.88083*	0.46657
CBR	−0.39808	0.41828	0.52121
SBR	0.13310	0.36599	−0.38729
PLR	0.87563*	0.52056	0.46535
KVR	0.57214	0.36988	0.62408
ULR	0.85522*	0.83779*	0.62254
LBR	0.44646	0.85525*	0.55029
HKR	0.00145	−0.11311	0.08385
PAR	0.54542	−0.02100	0.28942
VYR	−0.58008	0.51603	0.09925
SMR	0.15830	0.41940	0.16472
OLR	0.04901	0.19525	0.52937
ZLR	0.27109	0.71208*	−0.13321
MSR	0.76808*	0.83749*	0.84296*

^*^p < 0.05, bold–correlation over 0.8.

**Figure 7:**
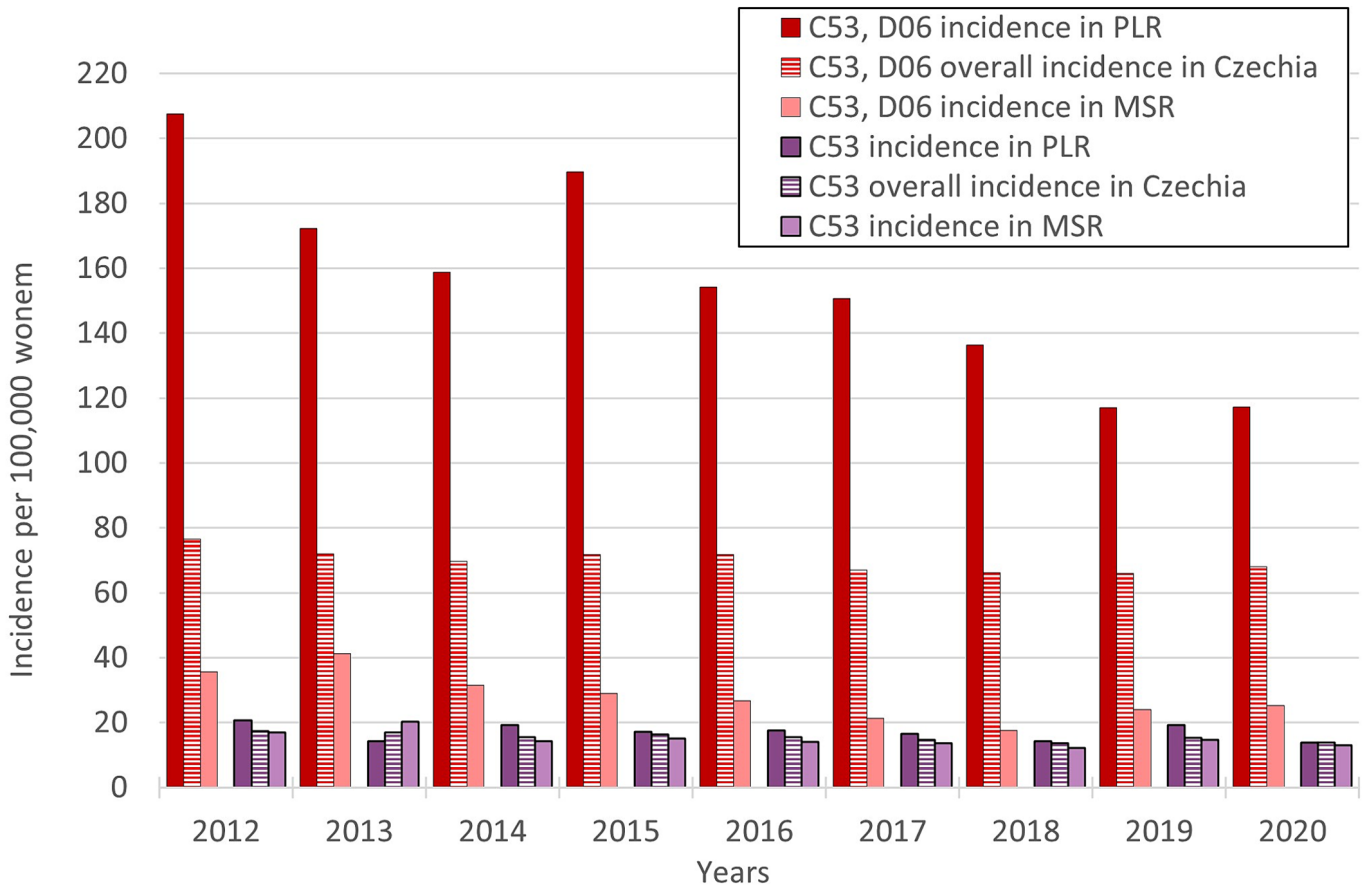
Trends of relative incidence of cervical tumours (C53, D06) in PLR and MSR in comparison to the overall trend of Czechia (PLR-region with the highest incidence of cervical tumours-C53, D06, MSR-region with the lowest incidence of cervical tumours-C53, D06).

**Figure 8:**
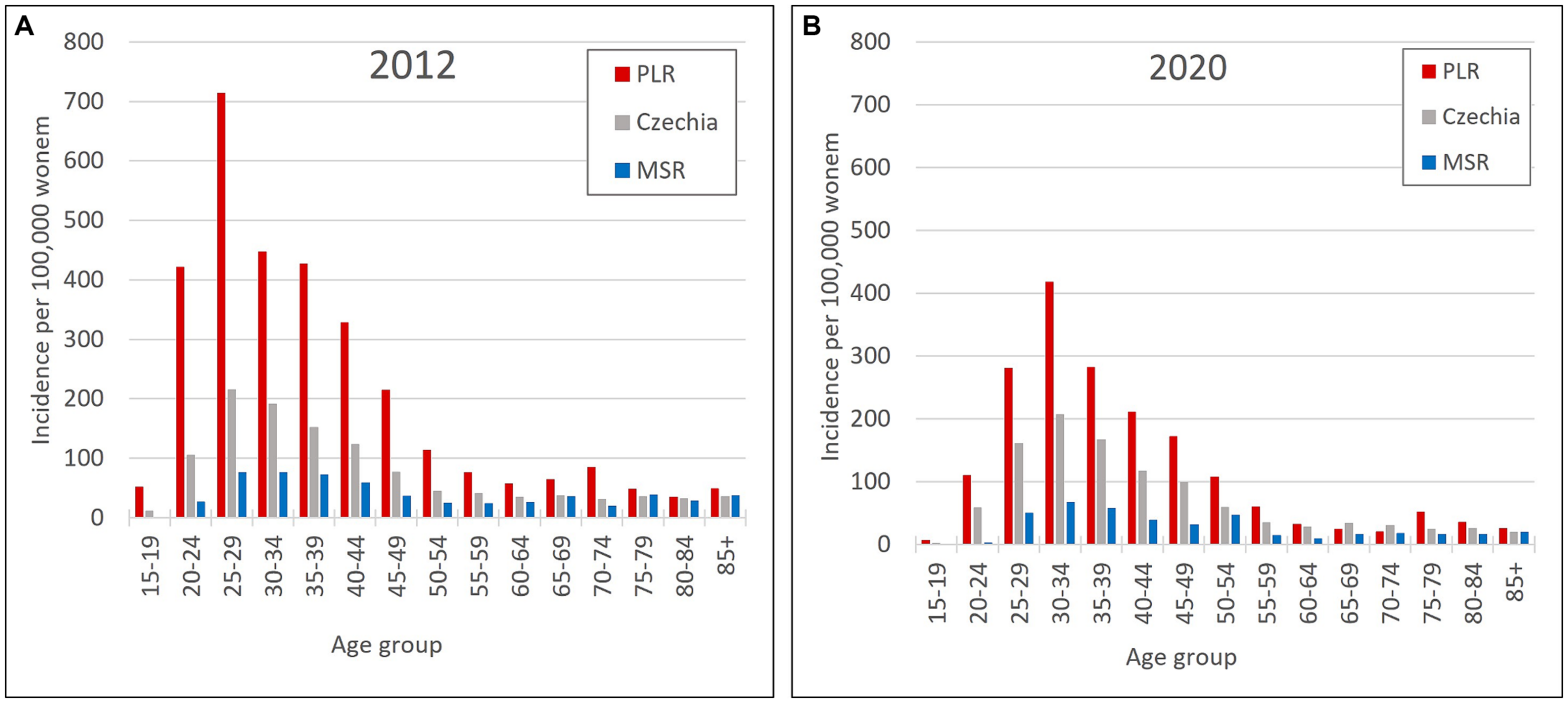
Incidence of cervical tumours (C53, D06) by age group in PLR (region with the highest incidence of C53, D06) and MSR (region with the lowest incidence of C53, D06) in comparison to the Czechia: **(A)** in 2012, **(B)** in 2020. (Incidences were calculated by dividing the absolute number by population size for each age group displayed as units available for 100,000 women).

**Figure 9:**
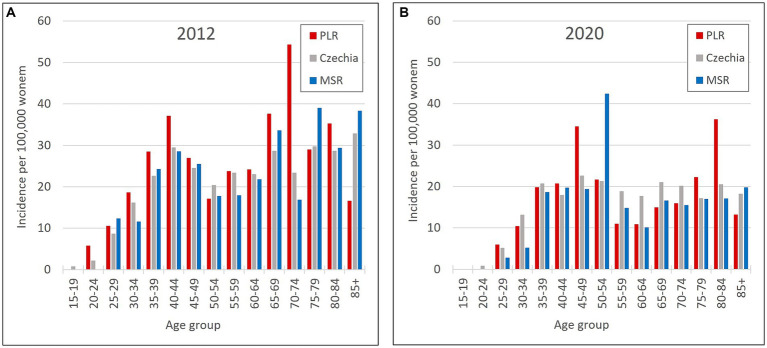
Incidence of malignant neoplasm cervix uteri (C53) by age group in PLR (region with the highest incidence of C53, D06) and MSR (region with the lowest incidence of C53, D06) in comparison to the Czechia: **(A)** in 2012, **(B)** in 2020. (Incidences were calculated by dividing the absolute number by population size for each age group displayed as units available for 100,000 women).

**Figure 10:**
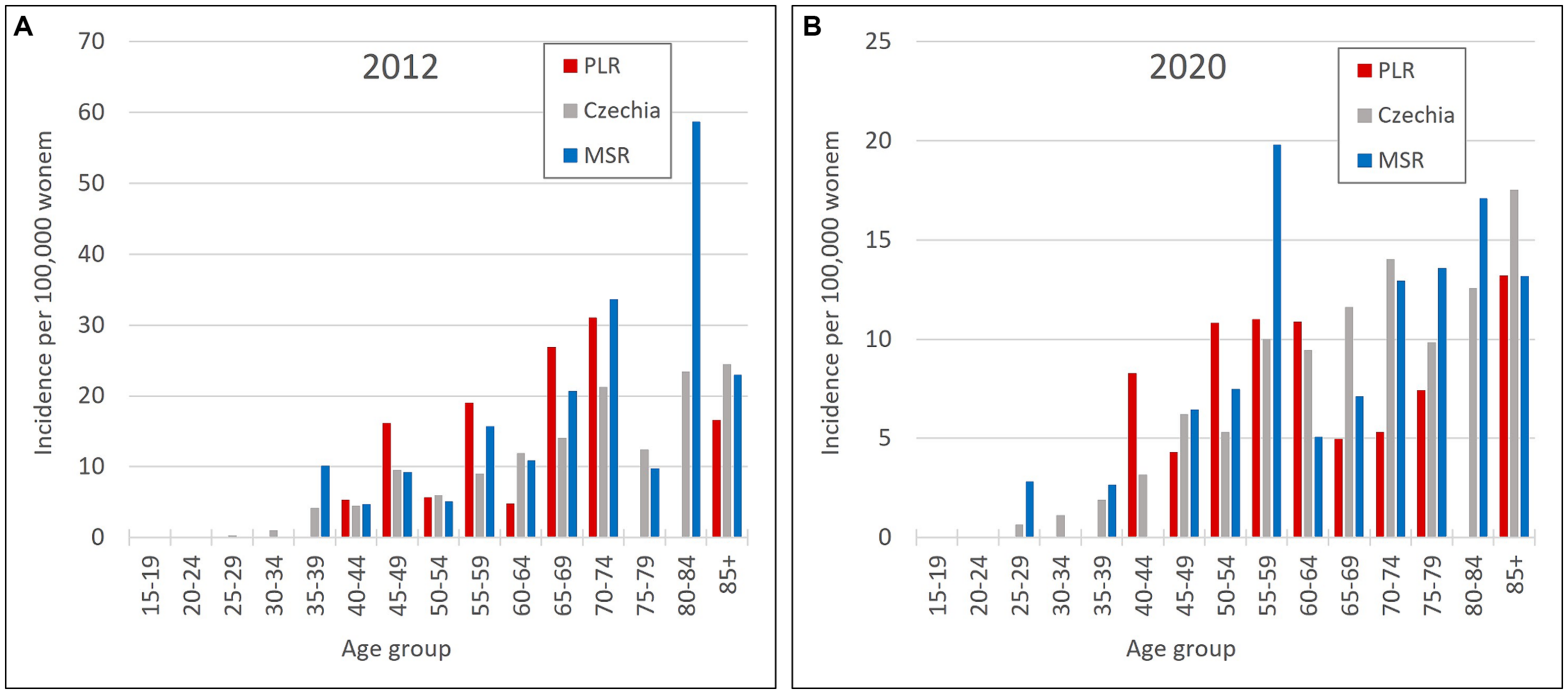
Mortality of cervical tumours (C53, D06) by age group in PLR (region with the highest incidence of C53, D06) and MSR (region with the lowest incidence of C53, D06) in comparison to the Czechia: **(A)** in 2012, **(B)** in 2020. (Mortality were calculated by dividing the absolute number by population size for each age group displayed as units available for 100,000 women).

So, the regions PLR and MSR are used for further analysis because of the similar trend with the overall trend of Czechia, but with diametrically different numbers of cervical tumours new cases through the studied period. Also, as already mentioned, the incidence of all cervical tumours (both malignant neoplasm C53 and carcinoma *in situ* D06) more closely reflects the risk of HPV exposure than separate malignant neoplasm C53. Trend of cervical tumours incidence in regions PRL - region with the highest cervical tumours (C53, D06) incidence and MSR-region with the lowest cervical tumours (C53, D06) incidence in comparison to the overall trend of the Czechia is shown in Figure 7. Figure 8 shows the incidence of cervical tumours (C53, D06) in selected regions in age groups. The relative incidence of cervical tumours (C53, D06) in PLR (the region with the highest incidence) decreased distinctly during the studied period. The most significant difference between 2012 and 2020 was observed in the most vulnerable age groups, the 20–44 age group. Figure 9 shows the incidence of malignant neoplasm cervix uteri (C53), where the difference is not apparent. Also, the difference between the two selected regions and the whole country is not prominent. Figure 10 shows the mortality of cervical tumours (C53, D06) in selected regions in different age groups.

### 3.3 The relationship of the cervical tumours incidence rate with selected demographic and socioeconomic indicators

Table 4 presents the results of the correlation analysis, focusing on demographic indicators of regions with the lowest (MSR) and highest (PLR) incidence in the period 2012–2020. The results show us the relationships between cervical tumours relative incidence and selected variables connected with the life and behaviour of women and, in some cases, the entire population. In the observed regions, there is a statistically significant positive correlation between cervical tumour relative incidence and (1) divorces (MSR, PLR), (2) abortions (PLR), (3) share of urban population (MSR, PLR), (4) the total number of students (MSR), and (5) women with primary education (MSR). These variables show positive correlations in both regions; however, they are not statistically significant in some cases. In both selected regions, we can observe a statistically significant negative correlation between cervical tumours relative incidence and (1) average age/age index (MSR, PLR), (2) live births woman (MSR, PLR), (3) average age of mother at birth (MSR, PLR), (4) marriages (MSR), (5) immigrants (MSR, PLR) and (6) woman with tertiary education (MSR, PLR). Regarding the regional aspects and differences, there are almost the same statistically significant results for the region with the lowest and highest incidence, except for population density. Focusing on data in more detail in PLR, there is an increase in population density and a decrease in the share of the urban population in the observed period. That explains the negative correlation between the incidence of cervical tumours and population density in this region.

**Table 4:** Correlation of cervical tumours relative incidences with demographic indicators of PLR (region with the highest incidence of C53, D06) and MSR (region with the lowest incidence of C53, D06) in 2012–2020.

Demographic indicators	Incidence of cervical tumours (C53, D06)		Incidence of malignant neoplasm cervix uteri (C53)	
	MSR	PLR	MSR	PLR
Average age	−0.83051*	−0.90066*	−0.73565*	−0.42274
Age index (in %)	−0.83439*	−0.90905*	−0.73625*	−0.44376
Total population gain/loss	−0.64035	−0.68537*	−0.47556	0.09011
Natural population gain/loss	0.06558	0.15789	0.12406	0.57649
Live births	−0.87465*	−0.50667	−0.79804*	−0.03280
Live births woman	−0.77059*	−0.68302*	−0.76025*	−0.32278
Live birth non-marital children	−0.89954*	−0.59018	−0.77441*	−0.29244
Average age of mother at birth	−0.80614*	−0.91391*	−0.74271*	−0.47224
Deaths	−0.26972	−0.45134	−0.33136	−0.64417
Marriages	−0.85092*	−0.48586	−0.63135	0.04953
Divorces	0.77123*	0.74145*	0.73698*	−0.02856
Abortions	0.61966	0.83304*	0.68225*	0.50334
Share of urban population^a^	0.78100*	0.88712*	0.70459*	0.45427
Population density^a^	0.79256*	−0.74552*	0.72604*	0.00253
Immigrants^a^	−0.86328*	**−0.84593**	−0.73900*	−0.25988
Emigrants^a^	−0.32974	−0.61883	−0.46769	0.42807
Total population change^a^	−0.22090	−0.60614	−0.03481	0.13745
Infant mortality rate^a^	−0.39217	0.25114	−0.04880	−0.58642
Students, total^a^	0.88551*	0.68642	0.70602*	0.20610
Primary education	0.81752*	0.59217	0.72776*	0.21121
Lower secondary education	0.58732	0.19336	0.42669	0.55877
Upper secondary education	0.66722*	−0.00347	0.55125	−0.17568
Tertiary education	−0.86722*	−0.71189 *	−0.71066*	−0.48460

*p < 0.05; ^a^indicators related to the entire population; bold–correlation over 0.8.

The following Table 5 presents the results of the correlation analysis, focusing on socioeconomic indicators of regions with the lowest (MSR) and highest (PLR) incidence in the period 2012–2020. The results show us the relationships between the relative incidence of cervical tumours and selected socioeconomic indicators. In the observed regions, there is a statistically significant positive correlation between cervical tumours relative incidence and the only socioeconomic variable–the unemployment rate of women (MSR). This correlation is positive in both regions; however, statistical significance is relevant only in MSR. In both selected regions, we can observe a statistically significant negative correlation between cervical tumour relative incidence and (1) gross domestic product (MSR, PLR), (2) the total number of pension recipients (PLR), (3) old age pensions (MSR, PLR), (4) average gross monthly wage per employee (MSR), and (5) employment rate (MSR).

**Table 5:** Correlation of cervical tumours relative incidences with socioeconomic indicators of PLR (region with the highest incidence of C53, D06) and MSR (region with the lowest incidence of C53, D06) in 2012–2020.

Socioeconomic indicators	Incidence of cervical tumours (C53, D06)		Incidence of malignant neoplasm cervix uteri (C53)	
	MSR	PLR	MSR	PLR
Gross domestic product^a^	−0.87380*	−0.90928*	−0.72644*	−0.38457
Pension recipients, total^a^	−0.34093	−0.80930*	−0.11113	−0.27193
Old-age pensions (single pensions)^a^	−0.89198*	−0.84954*	−0.74308*	−0.33473
Average gross monthly wage per employee^a^	−0.62028	−0.88669*	−0.55959	−0.41085
Unemployment rate (in %)	0.86352*	0.76345	0.76304*	0.28659
Employment rate (in %)	−0.90332*	−0.78131	−0.72180*	−0.47529

*p < 0.05; ^a^indicators related to the entire population; bold–correlation over 0.8.

## 4 Discussion

For many infectious diseases, women are at higher risk and have a more severe disease course than men for many reasons, including differences between biological and sociocultural factors. This study focuses on infections with human papillomaviruses (HPV) in the form of cervical cancer as a gender-specific disease. Before age 50, genital HPV infection occurs in 80 percent of women (8), and cervical cancer is the fourth most common cancer in women worldwide (7). Regarding scientific studies focused on cervical cancer and connected issues, there is still a gap in the knowledge of possible indicators which can influence both incidence and mortality.

Firstly, the overall trends of the incidence and mortality of cervical cancer in Czechia for the period 2012–2020 were observed. Generally, there is a decreasing trend in the incidence of cervical cancer in the observed period. Regarding cervical tumours (C53, D06), the decrease in incidence between 2012 and 2020 is approximately 11% (from 76.49 per 100,000 women in 2012 to 68.02 per 100,000 women in 2020). Focusing on the incidence of malignant neoplasm of cervix uteri (C53), the decrease is more than 20% (17.33 per 100,000 women in 2012 versus 13.82 per 100,000 women in 2020). In the case of mortality, the trend also shows a decrease in mortality rate since 2012, approximately 19% (from 6.67 per 100,000 women in 2012 to 5.40 per 100,000 women in 2020).

It is clear that vaccination is worthwhile, and the positive impact of vaccination on cervical cancer incidence and mortality should increase over time. Czechia has sufficient available vaccines and an established vaccination programme. On the other hand, the vaccination rate is decreasing despite financing the vaccines by insurance companies since 2012 for girls aged 13. Vaccination rates of girls aged 13 fell by 11.7% between 2012 and 2019 (from 75.5 to 63.9%). The leading cause of insufficient vaccination in Czechia is “vaccine hesitancy,” the distrust in vaccination caused by misinformation. For HPV vaccination, written informed consent from parents and children is needed. If the girl requests an offered HPV immunisation, but the parents refuse consent, she can be immunised. However, if the parents or guardians request immunisation, but the girl objects, a court decision is needed for being vaccinated (22). Overall, trust in vaccination in Czech society is decreasing. This applies to all types of vaccinations; overall, there is a decrease. This decrease is sometimes even more than 10% (e.g., MMR vaccine). The consequence of this behaviour is the occurrence of originally eliminated diseases. Support from policy-makers, the government, and ministries is necessary. Unequivocal support of primary prevention programmes, their accentuation and highlighting of benefits, the safety of these measures, etc. Unfortunately, the misinformation scene and fake news, which are related to vaccination as such, play a significant role in this.

On the other hand, the significant increase in the number of vaccinated boys, which was only 29.7% in 2018/19, is positive (23). According to the WHO, for example, Uzbekistan achieves high HPV vaccination coverage against cervical cancer when 94% of girls aged 12–14 are now covered with a first dose of HPV vaccine (33). Globally, about 50% of countries have introduced HPV vaccination. WHO issued a call for cervical cancer elimination in 2018 and recommended the extension of HPV vaccination to boys. HPV vaccination to boys appeared more cost-effective compared with increasing vaccine uptake amongst girls in cases where vaccination coverage amongst girls is persistently lower than 75–80%. Universal HPV vaccination is likely more effective and efficient in reducing HPV virus circulation in the general population, even at lower vaccine uptake levels. In December 2021, all European Union/European Economic Area countries introduced HPV vaccination in their national programmes. Several countries (i.e., Austria, Belgium, Croatia, Czechia, Denmark, Finland, France, Germany, Hungary, Ireland, Italy, Liechtenstein, the Netherlands, Norway, Portugal, Slovakia, Slovenia, Spain, Sweden, the United Kingdom) have extended, or have decided to extend in the coming years, HPV vaccination to boys (34, 35).

In Czechia, HPV vaccination for boys aged 13 has been covered by public health insurance since 2018. In neighbouring countries, vaccination strategies for boys differ. In Austria, the HPV vaccine is offered free of charge to all children aged 9–12 years since 2014. Before 2014, the vaccine was recommended but not publicly funded. In Germany, since November 2018, HPV vaccination for all 9–14-year-olds and catch-up HPV vaccination for girls and boys 15–17-year-olds has been included in the catalogue of mandatory benefits of statutory health insurance. In Poland, by 2021, HPV vaccination was not part of the mandatory vaccination programme but was recommended for boys and girls. In Slovakia, by 2021, both females and males were offered the vaccination and it is partially funded (34). According to the HPV information centre and its estimation for 2020 (24), in Czechia, an age-standardized incidence rate (ASIR)/age-standardized mortality rate (ASMR) of cervical cancer is 9.3/3.6 (per 100,000 women per year). In Austria, Germany, Poland, and Slovakia, ASIRs/ASMRs are 5.3/1.8, 7.6/2.2, 12.3/5.9, and 16.6/5.4, respectively. Czechia has the lowest ASIR/ASMR of the Eastern European countries. In Eastern, Western, Northern, and Southern Europe, ASIRs/ASMRs are 14.5/6.1, 7.3/2.1, 10.4/2.2, and 7.7/2.3, respectively (24).

Generally, it is evident that screening, vaccination, and the age of screening and vaccination are essential for decreasing both the incidence and mortality of cervical cancer (36). In Czechia, there is a noticeable decrease in the overall incidence of vaccination between 2012 and 2020. The most obvious decreasing trend is in all cervical tumours (C53, D06) aged 15–24 years. This reflects the targeting of the vaccination programme for girls aged 13 in Czechia, which started just in 2012 and is now manifested mainly in girls/women 10 years older. On the contrary, the highest incidence was in the age group 25–34 years, which was even over 200 per 100,000 women. In separate malignant neoplasm of cervix uteri (C53), the higher incidence (over 20 per 100,000 women) started appearing from the age group 35–39 years and above. Mortality increased significantly from the age of over 60 years, with the highest frequency at the age of over 85 years. According to a systematic review from 2022, in national immunisation programmes, most girls and boys are inoculated with the HPV vaccine by the time puberty begins; thus, it is essential to monitor the vaccine effect at least until the sexually active period in their 20s and 30s (37).

All Czech women over 15 years old are screened every year. For example, in a study from Italy (36), a bimodal shape in cancer incidence was observed, with a first peak in the 40–45 years age group, and a second, higher peak in the 75–80 years age group. Bimodality in cancer incidence was a consequence of the initiation of a screening programme covering the population only up to a given age (i.e., 70 years in Italy). In particular, the peak at high ages arises and is gradually magnified over time by the sudden increase of the population at risk of cervical cancer, which occurs at the exit of the screening age, contrasted with the cumulative success over time of diagnosis and treatment within the screened age groups.

Significant differences between regions can be observed in the incidence of cervical tumours and even in vaccination rates. For example, the HPV vaccination rate of prime-vaccinated female patients relative to the female population aged 13 years in regions of Czechia in 2019 varied from less than 56% in Prague (PCC) and ZLR region to more than 68% in OLR region and ULR region (see Figure 3). In some subregions of OLR region, the vaccination rate is more than 80% (21). Focusing on the cervical cancer incidence rate in the regions, in the case of all cervical tumours (C53, D06), the difference in the individual regions is evident (Figure 4). The region with the highest incidence in the whole analysed period 2012–2020 is PLR. On the contrary, the region with constantly lowest incidence is MSR. Regarding separate malignant neoplasm of cervix uteri (C53), there are also significant differences in the individual regions; however, no such regions consistently have the highest or lowest incidence numbers (Figure 5).

Concerning urban and rural areas, we can focus on 100% urban areas, such as Prague, the capital city (PCC). This region has the lowest vaccination rate and middle incidence rate both in all cervical tumours (C53, D06) and separate C53. In other regions, the yield of urban density varies. Urban and rural areas are divided by the number of inhabitants. A municipality of up to 3,000 inhabitants is considered a rural area, and above 3,000 as an urban area. Based on the results of correlation analysis, indicators connected with urban/rural aspects, such as a share of urban population and population density, are statistically significant. Regarding the variable “share of urban population,” there is a statistically significant positive correlation with cervical cancer incidence. Therefore, the locations with a higher share of the urban population show a higher incidence rate in Czechia. Comparing our results with other studies, regional differences, especially urban/rural differences, were observed in China (18). Also, in the United States (8), were observed geographic disparities in cervical cancer incidence, particularly in rural areas. Urban and rural disparities can influence access to healthcare resources. Rural residents may face challenges such as limited healthcare facilities or transportation issues.

Other important possible indicators that can influence incidence, apart from those mentioned above, are demographic and socioeconomic factors. So, the final incidence results from the simultaneous influence of all possible indicators. Identifying only the key variables with the most important impact is difficult. Focusing on the results of the correlation analysis presented in Tables 4, 5, there are indicators that can influence the incidence positively and/or negatively.

The indicators which can cause higher cervical cancer incidence are the high unemployment rate of women, the high number of divorces, the high number of abortions, the high share of the urban population, the high number of students, and the high number of women with only primary education. Such indicators are connected with economically disadvantaged citizens (women) and women from underprivileged families. It is also associated with the low level of education and living in urban places.

On the other hand, the indicators which can have a negative impact on cervical cancer incidence are the high GDP, the high average gross monthly wage per employee (indicates the economic level of a given region), the high employment rate of women, the higher average age of mothers at birth, and the high number of women with tertiary education. Such variables indicate the middle and upper class of citizens (women), women with higher qualifications and wages, and probably more heightened awareness connected with vaccination. Various case and expert studies (38, 39) have addressed the issue of prevention and the factors that influence the willingness of the population to undergo preventive health check-ups and/or vaccination. Their results support the hypothesis that socially and economically vulnerable people attend fewer preventive check-ups. They usually lack information about vaccination or may be misinformed. For example, Brunner-Ziegler et al. (38) focused on participation in preventive health check-ups in Austria. Regarding the variables, middle-aged participants, had secondary education (women) or tertiary education (men), higher income, and were born in Austria (men) or another member state of the EU-15 (women) were more likely to have undergone a preventive health check. Another study from Germany (39) underlined the important influence of socio-economic indicators, such as education, occupation, and income.

Focusing on other scientific studies, our results underline the problem of economically disadvantaged regions and families. For example, Kapoor and Sharma (16) identified the following risk factors in India: early age at marriage, lack of education, increased parity, early age at first pregnancy, poor sanitation, use of tobacco, and belonging to below-the-poverty line. Buskwofie et al. (7) depicted the following risk factors in the USA: racial and ethnic minorities and socioeconomically disadvantaged. Concerning the situation in China, Lin et al. (17) observed the joint effects of various socio-demographic factors, including residence, education and monthly income. Except for age, residence, education, monthly income, number of sexual partners in the past 6 months, and parity, the authors noticed that high HPV knowledge level was significantly associated with HPV testing behaviour.

Due to the amount of data, it is not possible to show all mentioned indicators in Czechia in a spatial context, and it is not even the current aim of the presented study. However, how the socio-economic indicators look in this context can be found, for example, in the Statistical Atlas administered by the Czech Statistical Office (40).

Our indicators analysis is concepted as ecological epidemiological study, so causal relationships cannot be inferred. But, it can help to understand the relationship between evaluated indicators and the incidence of cervical tumours. Unique is the focus not only on the incidence of malignant cervical tumours but also on the incidence of carcinoma *in situ*, which both together more closely reflect the risk of HPV exposure. Also, the location of Czechia in the centre of Europe and its regional diversity enables the transferability of the results to other similar regions and contributes to the development of knowledge in this area. The follow-up research will focus on a more detailed analysis of significant indicators in a spatial context, a series analysis of trends, more complex multivariable analyses and potential confounders.

According to the results and our findings, we can support already published statements and outcomes (14, 41–43): (i) vaccination against HPV has been demonstrated to reduce the risk of HPV-related diseases effectively; (ii) vaccination primarily aims to prevent cervical cancer (common vaccines include Gardasil and Cervarix, which protect against the most prevalent cancer-causing HPV types); (iii) administering the HPV vaccine before exposure to the virus, which typically occurs usually through sexual activity, is most effective (it is recommended around age 11 or 12); (iv) despite vaccination, regular cervical cancer screening (such as Pap smears or HPV testing) remains crucial for detecting and treating any pre-cancerous changes.

Education in the prevention of HPV through vaccination is essential. So does accentuation of the preventive programmes for both primary and secondary, as well as tertiary prevention. Ensuring the interest of the general population in the issue of HPV and the possibility of their prevention, which requires the cooperation of the media and policy-makers, is also essential. Last but not least, there is a need for education in the field of fake news regarding the usefulness and safety of preventive measures.

## 5 Conclusion

HPV continues to be a significant public health concern. Despite improvements in cervical cancer screening and treatment, the incidence of cervical cancer (C53) in Czechia remains relatively high, with 13.8 cases per 100,000 women in 2020. This underscores the need for increased awareness and prevention efforts, including vaccination and regular screening. Although the HPV vaccine is available and recommended for both boys and girls in Czechia, vaccination rates remain relatively low. As of 2019, only 63, 9% of girls aged 13 had the HPV vaccine. Increasing vaccination rates could help to reduce the burden of HPV-related diseases in the country.

In summary, while there have been some improvements in the prevention and management of HPV-related diseases in Czechia since 2012, there is still much work to be done to reduce the prevalence of HPV and the incidence of related cancers. Increasing vaccination rates and promoting regular screening for cervical cancer should be priorities for public health efforts in the country as well as the influence of vaccination on the decrease in the incidence rate of cervical cancer is significant, and it would be worthy to support awareness in the population, especially in regions with higher incidence rates.

There were observed differences between regions. Results underline the problem of economically disadvantaged regions and families. Based on correlation analysis, indicators connected with urban/rural aspects, such as a share of urban population and population density, were statistically significant. The indicators related to higher cervical cancer incidence are the high unemployment rate of women, the high number of divorces, the high number of abortions, the high share of the urban population, the high number of students, and the high number of women with only primary education. On the other hand, the indicators which are related to lower cervical cancer incidence are the high GDP, the high average gross monthly wage per employee, the high employment rate of women, the higher average age of mothers at birth, and the high number of women with tertiary education.

## Data availability statement

Publicly available datasets were analyzed in this study. This data can be found here: For incidence and mortality of cervical tumours in the Institute of Biostatistics and Analyses repository, http://www.svod.cz. For data about the age distribution of the women population in the Czech Statistical Office repository, https://www.czso.cz/csu/czso/age-distribution-of-the-population-2021. For data about territorial comparison of demographic and socioeconomic indicators in the Czech Statistical Office repository, https://www.czso.cz/csu/xm/mezikrajske_srovnani_vybranych_ukazatelu. For data about the territorial comparison of demographic and socioeconomic indicators only about women the Czech Statistical Office repository, https://vdb.czso.cz/vdbvo2/faces/en/index.jsf?page=uziv-dotaz. For geographical data in the State Administration of Land Surveying and Cadastre repository, https://geoportal.cuzk.cz/(S(gbqevdvghr43bw5iyj41kpss))/Default.aspx?lng=EN&menu=2291&mode=TextMeta&side=mapy_data200&metadataID=CZ-CUZK-DATA200-HRANICE-V.

## Author contributions

OH: Conceptualization, Methodology, Supervision, Writing – review & editing. OM: Conceptualization, Data curation, Formal analysis, Methodology, Writing – original draft. TS: Data curation, Software, Visualization, Writing – original draft. DN: Data curation, Methodology, Writing – original draft. JZ: Conceptualization, Data curation, Methodology, Supervision, Writing – review & editing. RK: Conceptualization, Writing – original draft. JV: Conceptualization, Methodology, Writing – review & editing.
